# Developing Green Innovations in the Wine Industry: An Applied Analysis

**DOI:** 10.3390/foods12061157

**Published:** 2023-03-09

**Authors:** Eduardo Sánchez-García, Javier Martínez-Falcó, Antonio Alcon-Vila, Bartolomé Marco-Lajara

**Affiliations:** Department of Management, Faculty of Economic and Business Sciences, University of Alicante, 03690 San Vicente del Raspeig, Alicante, Spain; javier.falco@ua.es (J.M.-F.); antonio_a_v_12@hotmail.com (A.A.-V.); bartolome.marco@ua.es (B.M.-L.)

**Keywords:** green innovation, green transformational leadership, green motivation, green creativity, wine industry

## Abstract

Winemaking is an ancestral activity characterized by its strong roots in the culture, heritage, and people of the producing regions. In addition to providing important health benefits, wine is a product that is widely accepted by society. However, the socioeconomic context is evolving at a rapid pace, and new requirements and needs are forcing companies to innovate in order to remain competitive in the markets, especially in terms of sustainability. The main aim of this paper is to analyze the relationship between green transformational leadership and green innovation, as well as the mediating effect of green motivation and green creativity in this relationship. Data were obtained from a sample of 196 Spanish companies belonging to the wine industry and, as a method of analysis, was used partial least squares structural equation modeling PLS-SEM. The results show a positive and significant relationship between green transformational leadership and green innovation in wine companies. Furthermore, green motivation and green creativity exert a mediating effect in this relationship. It is concluded that the managers of wine companies should encourage employee motivation and creativity, especially in ecological terms, by creating an environment conducive to the development of environmentally friendly innovations.

## 1. Introduction

The negative impact of human action, especially of business activity, on the natural ecosystem, puts the sustainability of our current socioeconomic development model at risk [1]. In recent years, there has been a growing trend among businesses and scholars to include a sustainable approach in their processes and actions. This trend has been driven by a variety of factors, including consumer demand, brand differentiation, regulatory pressures, cost reduction, and a growing recognition of the need to address environmental and social challenges. In this vein, stakeholders and academics have exerted pressure on organizations to formulate social, economic, and environmental policies [2,3]. Recent studies have shown, for instance, that greening information technology and computing processes may assist firms in minimizing carbon emissions, the use of energy, and waste removal operations [4,5]. Thus, the attention of researchers has increased towards the introduction of an environmental perspective in each of the areas or departments that make up the companies, being essential to promote creativity and the development of innovations within the firms [6,7]. Increasingly, businesses seek to encourage environmentally responsible practices and develop environmentally conscious brands. Greening approaches to business are based on the organization’s environmental knowledge, leadership, and capabilities, which, from an ecological perspective, results in a functioning corporate governance structure and improved operating performance [8]. In this manner, industry emphasis has shifted towards a greener approach in a variety of sectors, with innovation, leadership, motivation, and creativity being some of the most crucial [9].

In the field of corporate sustainability, several scholars have established that a company’s attitude towards fostering creativity and innovation for environmental purposes can play a key role in improving resource efficiency and its reputation in the marketplace, as well as the firm’s performance both financially and strategically [10,11,12]. Creativity is generally recognized as a predictive factor of organizational performance enhancement, competitiveness, and customer service activities [13]. In this way, companies can apply green creativity to the development of new technologies, systems, or techniques to reduce environmental degradation [10,14,15]. Furthermore, by leveraging their green creativity, businesses may become more innovative, which can help them to improve their competitive position in the markets [16]. Green creativity has emerged as an individual cognitive capacity for implementing solutions that are environmentally responsible [17] and concerns innovative and valuable ideas about environmental goods, services, or activities, which are influenced by individual and collective elements [10,15,18]. In connection with this, policymakers seem to be increasingly aware of the potential of environmental innovation to combat climate change, which may influence their support for the development of new solutions by companies to reduce the impact of business activity on the natural environment [10]. As a result, there seems to be a tendency for companies to develop innovations to reduce the impact of their activities on the environment [17]. Nevertheless, future research is required to comprehend the function of managers in ensuring the sustainability of firms and, particularly, the role of transformational leadership [19,20].

Firms’ success is contingent on their ability to use resources that are essential, uncommon, and costly for competitors to replicate [21]. The resource-based perspective posits that a company’s capacity to harness its strategic resources may drive sustained performance and boost competitive advantage [22]. From this viewpoint, green transformational leadership is found to be a crucial resource for firms’ environmental performance [23]. Several researchers examined how green transformational leadership, environmental management systems, and a company’s performance are related, concluding an indirect impact that materializes through various factors and practices [3,19,20,24]. Green transformational leadership may be defined as the conduct of managers that encourage their subordinates to meet environmental objectives, inspiring them towards surpassing the expected levels in this respect [10]. This kind of leadership encourages eco-friendly innovative behavior among workers, which can positively influence the efforts made by companies to reduce the environmental impact of their activities [20,25]. Previous evidence suggests that, from an environmental perspective, leadership can be a relevant factor in driving creative behavior [14,24,26]. Furthermore, those leaders with a positive image regarding the environmental management strategy of the firm demonstrate respectful conduct towards the environment and welcome innovative thinking that promotes the achievement of the corporate environmental conservation goals [18].

However, not only creativity but motivation may have a significant effect on a company’s level of innovativeness. Motivation is inherently pleasurable and is effective when individuals or workers believe that it has an internal origin [27]. In this vein, there is widespread concern about the protection of the natural environment, which tends to motivate people to act, to a greater or lesser extent, in favor of its preservation. Respect for the environment seems to be an intrinsic human motivation, which may be translated into efforts dedicated to the development of solutions that protect and preserve the natural heritage from damage caused by human activity. In this respect, employees can come up with more creative eco-friendly ideas by having a greater desire to produce environmentally friendly goods.

Regarding the relevance of this research, understanding the way in which transformational leadership drives motivation, creativity, and innovation within firms is essential to know how organizations can promote and sustain innovation in today’s rapidly changing business environment. Transformational leadership is a leadership style that emphasizes inspiring and empowering employees, fostering a positive organizational culture, and encouraging innovation and change. Then this kind of leader can have a positive impact on employee motivation, creativity, and innovation, inspiring and motivating them to work towards a common vision and promoting a sense of ownership and commitment among employees that can drive creativity and innovation. They also can drive the creation of a positive work environment that fosters open communication, collaboration, and risk taking, all of which are important for innovation. By providing employees with the necessary resources and support, such as training, feedback, and recognition, transformational leaders can create an environment that promotes innovation and creativity, that is, building a culture of innovation within the organization, promoting experimentation and the pursuit of innovative ideas.

However, few studies from an environmental approach have turned their attention to green innovation from the executives’ and workers’ standpoint [14,18]. Empirical evidence is lacking in the literature on how green transformational leadership influences green motivation and creativity, and how these characteristics affect green innovation [17,24]. The main objective of this research is to analyze the relationship between green transformational leadership and the development of green innovations within firms. Furthermore, it is intended to determine whether there is a mediating effect of green motivation and green creativity in the established relationship. Then, it is intended to address whether green transformational leadership, directly and indirectly, influences firms’ green innovative performance, using the specified mediating variables. This paper focuses its attention on the wine industry, which manufactures products of great market value and is particularly affected by accelerated climate change caused by human action, especially due to the potential negative impact of business activity on the environment [1,28,29,30]. Then, this research can provide valuable insights into how organizations can effectively promote green innovation and sustainability while fostering employee motivation and creativity by driving green transformational leadership.

The structure of this paper is as follows. After this introduction, the following section delves into the literature that serves as the basis for the approach of the hypotheses. The third section explains the methodology, and then the results of the statistical analysis are exposed and discussed. Finally, the conclusions, limitations, and possible future lines of research are drawn.

## 2. Literature Review

### 2.1. Green Transformational Leadership and Green Innovation

In recent decades, rapid economic expansion has led to unsustainable exploitation of available resources and large-scale waste generation, with the consequent negative impact on the natural environment. In this regard, numerous researchers have pointed out in the last few years the need to develop and implement strategies to minimize the degradation of the natural ecosystem as a result of business activity [31,32]. Innovation is a key factor to produce healthy and high-quality products, especially in the food and beverage industry, as well as to enhance firms’ competitiveness, even in traditional ones, such as the wine industry [33,34]. However, from an environmental point of view, innovation is a factor that allows companies to reduce their environmental impact by developing and implementing less hazardous techniques, systems, or manufacturing processes [35,36]. Green innovation may be understood as the successful development and implementation of a product, process, technique, or system that reduces the environmental impact of businesses, which can have several advantages for firms, including reputation enhancement, cost savings, and the ability to respond to environmental challenges [37,38]. Thus, green innovation, in addition to enabling companies to address environmental problems and promote sustainable development, is conducive to superior performance [25,39].

Green innovation promotes economic expansion and progress, being a relevant element in overcoming the current environmental challenge [40,41]. Multiple experts have asserted that green innovation is an essential precursor to sustainable development [42,43] but provide inconclusive findings [7]. This kind of innovation involves modifying goods, processes, and technology to limit environmental damage and maximize efficiency in the use of resources, which allows businesses to cut manufacturing costs and timespan, among other benefits [44]. In addition, these innovations promote the attraction of new consumers willing to spend more money on environmentally responsible services and goods, hence enhancing the businesses’ productivity and profitability [17,30,43]. Despite the evidence of the influence of green transformational leadership on sustainable development, there is still a need to deepen its role in the generation of green innovations [25,38]. Considering the current business ecologism movement, the development of environmentally friendly products and practices are becoming crucial aspects for managers [7].

Regarding this, it has been acknowledged that green transformational leadership is a significant element influencing green product creation [43]. This notion relates to inspiring and motivating enterprises to fulfill their environmental objectives [10]. According to Gerini et al. [45], people change their behavior when facing crises, so employees may adapt their attitude toward climate change if they are more aware of the importance of reducing the negative impact of the firms on the natural environment. In this vein, green transformational leadership instills green objectives into the view of the company and pushes employees to absorb and implement sustainable attitudes and ethos to accomplish organizational and environmental objectives [24,25]. Moreover, it is a crucial factor in attaining environmental sustainability because it encourages people to think outside of the box, establish unique connections, and promote green policies and practices to drive innovation in this area [2,46]. According to Xie et al. [44], this leadership style enables the generation of green innovations through the integration of current industry information and tendencies and the latest technologies, as well as raising funds and employee training in innovative processes, among others. Firms’ knowledge base in this regard seems to positively affect their environmental performance [47].

Numerous studies show that green transformational leadership contributes to limiting the negative consequences of industrial pollution on the surrounding environment [2,48]. In this vein, recent discussions establish that green transformational leadership may enhance the pursuit of green performance through the development of green innovations [24,25,49]. Nevertheless, to date, there is a limited understanding on how companies can benefit from green transformational leadership [24]. On the basis of the above, the following hypothesis is formulated.

**Hypothesis 1** **(+).** *There is a positive and significant relationship between green transformational leadership and firms’ green innovation*.

### 2.2. Green Motivation

Motivation may be defined as a condition in which goal-directed actions are sustained by people’s love and enthusiasm, in addition to other personal incentives and advantages [50,51]. This is the core ingredient of creativity that keeps people committed to their professions, heightens their concentration, and, consequently, leads to greater contentment, expertise, and innovative behavior [52,53]. The capacity for feeling and thinking in terms of the company’s environmental sustainability drives employees to carry out green actions, so inspiring them to reach specified environmental objectives [54]. Norton et al. [55] emphasized the significance of motivation in fostering green innovation practices inside an organization. When workers are in a favorable emotional state, they are more motivated to attain their own objectives, including environmental goals [56].

Green transformational leaders may serve as a positive role model for workers, enabling them to better internalize the ideals and objectives of environmentally sustainable development, strengthening their autonomous drive to engage in environmentally friendly behaviors [57,58]. As workers adopt and internalize the values communicated by their leaders, the significance of such environmental values in their creation of self-identity will increase, making environmental protection efforts more meaningful [59,60]. In this way, green motivation occurs when companies become environmentally conscious and encourage employees to generate ideas and plans for greening the company in order to maintain the surrounding natural ecosystem [17,61]. Green motivation has prompted companies to reconsider the goods they produce and the processes they use to manufacture them, therefore stimulating the creation of environmentally sustainable solutions [17].

Green transformational leaders drive the motivation of environmentally responsible employees through professional development and the establishment of common values [25,62]. Then, these kinds of leaders impact the beliefs, values, and ideas of followers by communicating their vision of a greener future through corporate actions and environmentally responsible innovation development [43]. This distinctive leadership style contributes to the creation of an atmosphere conducive to increasing motivation from an environmental standpoint. Green transformational leadership imbues employees with new beliefs, values, and competencies, and, in return, they increase dedication and engagement, which may result in higher creativity and an improvement in the firms’ environmental performance [10,63]. Previous research examined the link between the traditional conceptions of transformational leadership and motivation, finding a favorable correlation among them [64,65].

Then, under green transformational leadership, people may be driven to carry out green activities and engage in pro-environmental behavior, endowing environmental duties with their own significance via leaders’ vision, charisma, and inspiration [10]. In this sense, stimulation and support from managers, linked to personal recognition, can enhance commitment and willingness among employees to engage in environmental tasks, being the moral and technical support provided by managers, which is a key factor that increases the motivation of their employees to carry out research and progress in environmental matters [10,25]. This leads us to offer the following hypothesis.

**Hypothesis 2** **(+).** *Green motivation exerts a mediating effect on the relationship between green transformational leadership and firms’ green innovation*.

### 2.3. Green Creativity

The act of creativity is a simultaneously psychological, social, and physical phenomenon, and provides novel ideas and potential solutions [66,67]. The atmosphere created in the workplace may either encourage or inhibit the enthusiasm and inventiveness of the company’s employees [68]. The fact that companies allow their employees to develop skills linked to an area relevant to the organization can favor the increase of creativity, both individual and organizational. In this way, creativity can be trained and developed so that through practice, the creativity of the employees and the company can be boosted [68,69]. In this vein, motivation may be seen as the core premise for creativity and, therefore, as the driving force behind their innovative conduct for workers to be more creative when they perceive a task to be interesting, engaging, and challenging [68]. Various studies suggest some external elements that can enhance creativity, among which it is worth highlighting the support of well-established leaders and an innovative atmosphere [70,71,72].

Transformational leaders exhibit unusual and innovative conduct and serve as an example of innovation throughout the business, having a crucial role in generating original ideas and producing innovative results [25]. A leadership sensitized to environmental sustainability may drive the motivation of the company’s employees in this area, which can lead to an improvement in the environmental performance of the organization [73]. In this way, they are able to motivate team members and raise subordinates’ knowledge on fundamental issues of the coexistence and respect for the environment [74]. Through the implementation of an inspiring leadership, employees can be encouraged to think creatively, enabling them to observe things from new perspectives and acquire new valuable knowledge [75,76]. Therefore, personalized attention from charismatic leaders makes it possible to discover the unique needs and motivations of each employee, and communication can be individualized to increase their engagement with the organization’s objectives [77].

Green transformational leadership stimulates employees to prioritize corporate objectives above personal ambitions, directs them in all circumstances, aids them when necessary, and inspires them to produce unique environmental solutions [25]. In this way, through the establishment of effective leadership that generates a positive, collaborative, and challenging climate in the company oriented towards innovation, the generation of novel ideas by all members of the company is motivated so that, through practice, employees improve their creative abilities [78]. Thus, green transformational leaders must advise and encourage their staff to engage in green innovation [10,63]. Green creativity may be interpreted as novel and valuable ideas aimed at the development of green goods, services, processes, or behaviors [10]. By recognizing workers’ requirements, skills, and incentives, this kind of leadership encourages people to offer green insights applicable to the firm. Indeed, this enables them to think out of the box, study problems from a variety of perspectives, and investigate novel solutions to environmental challenges [10], and may drive employees to seek novel ways to their responsibility on ecological issues. Given the above, the following hypothesis is proposed.

**Hypothesis 3** **(+).** *Green creativity exerts a mediating effect on the relationship between green transformational leadership and firms’ green innovation*.

### 2.4. Effect of Green Creativity on the Development of Green Innovations, Driven by Organizational Environmental Leadership and Motivation

Green transformational leadership enables companies to adapt increasingly better to the requirements of the natural environment, freeing them from organizational inertia and opening up new paths for development. [2,38]. This kind of leadership focuses on long-term objectives, fosters positive values among employees, and organically motivates the staff to acquire environmental competencies [63,79]. Then, these leaders motivate staff to produce new information and alternative ideas and to build the comprehension and application skills necessary to use them [80].

Such leaders provide an atmosphere that inherently stimulates employees to participate in tough, non-routine tasks and makes the work more exciting through creative thinking and innovative approaches to solving business challenges [81,82]. In this view, an employee’s drive to complete creative tasks improves when the activity is attractive, demanding, fun, and intriguing [78]. Transformational leaders committed to the environment can improve the company’s results in this area because, by knowing and meeting employees’ requirements individually, they can encourage creative thinking among them and increase their engagement in reaching the company’s environmental goals [49]. In addition, they may influence organizational innovation, hence fostering the development of green practices inside businesses [14,83]. In this regard, it is important to remember that creative behavior should be managed and directed towards problem solving; otherwise, the company would be unable to respond effectively to environmental difficulties [47,84]. The participation of creative people is essential to boost the probability of achieving green innovation by the company [35,39,85].

**Figure 1:**
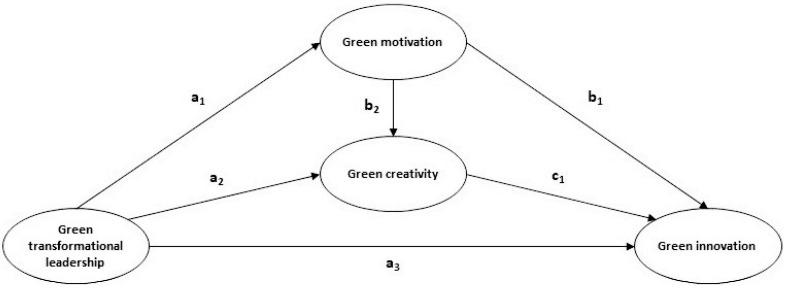
Nomogram of the proposed model. H1 = a3: Green transformational leadership → Green innovation. H2 = a1 × b1: Green transformational leadership → Green motivation → Green innovation. H3 = a2 × c1: Green transformational leadership → Green creativity → Green innovation. H4 = a1 × b2 × c1: Green transformational leadership → Green motivation → Green creativity → Green innovation.

Employees that lack enthusiasm, passion, and interest in performing green creative tasks probably do not provide the desired results in environmental terms [27,86]. Green motivation may only be effective if workers are interested in ecological concerns. Some personnel may have a greater appreciation and enthusiasm for the natural world and might be more inclined to engage in ecological activities. In this vein, supportive leadership generally demonstrates concern for the needs and emotions of employees, helping them to develop their talents, so if also committed to minimizing the environmental impact of the firm, then they can foster personnel motivation and creativity in these areas [87,88]. Furthermore, the trust of the leaders in their staff may stimulate risk-taking skills and the ability to think critically [25]. The notion of creativity suggests that people are more creative in a context in which leadership encourages personnel to see their activities as inspiring, enjoyable, interesting, and difficult [68]. Therefore, in the business environment, the role of green transformational leaders is to drive an eco-friendly motivation that fosters a creative green mindset among employees [78,89]. On the basis of the above, it is developed the fourth hypothesis, and the nomogram of the model is showed in Figure 1.

**Hypothesis 4** **(+).** *There is a double mediation of green motivation and green creativity in the relationship between green transformational leadership and firms’ green innovation*.

## 3. Materials and Methods

### 3.1. Population and Sample

The population under examination consists of Spanish enterprises operating in the domain of wine industry. According to the SABI database, in 2021, there were 4373 firms functioning in Spain, of which around 99% were MSMEs. The sample includes 196 operational Spanish firms. Spain is one of the world’s leading wine producers, with a long history of wine production and a diverse range of wine regions. This country has the largest vineyard area in the world, accounting for 13% of the world’s total surface area (more than 941,000 hectares of vineyards), is also leader in organic vineyards (more than 121,000 hectares of organic vineyards), the world’s leading exporter by volume (more than 2.3 billion liters), and the third largest exporter by value (2914 million euros). In addition, more than 85% of the Spanish wineries export their products to 189 countries. This industry employed 2.4% of the overall workforce in Spain and contributed 2.2% of the gross added value in 2021 (information extracted from the website of the Spanish Wine Federation, https://www.fev.es/es/ (accessed on 28 February 2023)).

Spanish wine industry has a strong focus on quality and sustainability, which has enabled it to build an excellent reputation at international level and positioned itself as a major contributor to the country’s economy [90]. Spain has over 60 wine regions, each with its own unique climate, soil, and grape varieties, which allows Spanish winemakers to produce a wide range of wines [91]. According to this author, Spanish wines are known for their high quality, and several wine regions, such as Rioja and Priorat, have gained international recognition for their distinctive styles, which has positioned Spain as a leading producer of premium wines at international level. In recent years, the Spanish wine industry has also seen an increase in organic and biodynamic wine production.

This trend reflects growing consumer demand for sustainably produced wines and a recognition of the environmental benefits of organic and biodynamic agriculture. Despite these strengths, the Spanish wine industry faces several challenges, including competition from other wine-producing countries and changing consumer preferences [90]. Spanish companies in the wine industry share several key features, including their focus on traditional production methods, their use of local grape varieties, and their emphasis on wine tourism as a key driver of growth. Many Spanish wineries have been in operation for centuries and continue to use traditional techniques such as hand-harvesting, foot-treading, and aging wine in oak barrels [92]. These methods are seen as essential for preserving the unique character and quality of Spanish wines. In this vein, Spain has a wide range of grape varieties, many of which are unique to the country, and Spanish winemakers often prioritize the use of local varieties in their wines [92]. This emphasis on local varieties helps to distinguish Spanish wines from those produced in other regions and adds to the diversity and complexity of the Spanish wine industry.

### 3.2. Data Collection and Measurement of Variables

By creating and distributing a questionnaire, which is shown in Appendix A, data were collected. After evaluating the statistical validity of the completed questionnaires and deleting those deemed invalid (due to a substantial amount of lost data, patterns of response, or single-value responses), 196 valid replies were obtained. Through their “minimum R^2^” technique, Hair et al. [93] showed that a model with a minimum determination coefficient (R^2^) value of 0.500 and a maximum of 3 predictors requires a minimum sample size of 38.

Green transformational leadership (independent variable) was measured by a seven-point Likert scale. It was built based on the study of Chen and Chang [10] and has six components. Green innovation (dependent variable) was evaluated using a seven-point Likert scale and has eight items. Based on the study of Chen [16], validated scales comprised four items, each used to assess the innovative performance of the product and process, respectively. Green motivation (mediating variable) was assessed using a seven-point Likert scale. It was built based on the research paper of Úbeda-García et al. [94] and has four items. Green creativity (mediating variable) was measured using a seven-point Likert scale. It was built based on the study of Chen and Chang [10] and Song and Yu [18], and has six items.

### 3.3. Analysis Technique

To evaluate the hypotheses, we used the second-generation multivariate technique of partial least squares structural equation modeling, PLS-SEM, and, particularly, the software SmartPLS was employed (version 3.9; company SmartPLS GmbH, Oststeinbek, Germany). This is a statistical technique used to analyze the relationships between latent constructs in a dataset. A great number of researchers in the field of strategic business management have put their focus on this technique [95].

According to Hair et al. [96], this method is appropriate for predictive analytics, particularly in social sciences, due to the latent character of the variables considered in this field. In addition, it is a flexible approach to modeling complex relationships, estimates the relationships between latent constructs and manifest variables directly, and allows for the testing of both reflective and formative measurement models [97,98,99]. Moreover, it is an efficient tool for the estimation of complex models with a large number of latent variables measured by multiple indicators or various structural relationships and facilitates the modeling of the relationships between a large number of variables, offering greater flexibility and robustness than traditional approaches [96].

## 4. Results

To assess the measurement model, it must investigate its internal consistency and convergent and discriminant validity [96]. Dijkstra-rho Henseler’s (ρA) is utilized according to these authors. As seen in Table 1, every outcome is significantly greater than 0.7 [96,97,98].

To assess internal consistency, besides analyzing Dijkstra-Henseler Rho A, Cronbach’s alpha and composite reliability were examined. Regarding the evaluation’s convergent validity, the measurement is performed by evaluating the reliability of the indicators, i.e., the size of the external loadings (λ) and the Average Variance Extracted (AVE), which refers to the total mean value of the squared loadings of the indicators belonging to the same construct [96]. The external loadings have a value greater than 0.707, and the AVE is higher than 0.5; therefore, this requirement is also met [96,98].

Historically, cross-loading analysis and the Fornell and Larcker method have been used. Nevertheless, the Heterotrait–Monotrait Ratio (HTMT) is a more effective tool for determining discriminant validity difficulties [99]. Kline [100] states that the HTMT ratio must be less than 0.85. The model largely satisfies this criterion, as demonstrated by Table 2.

### Structural Model Assessment

The evaluation of the structural model helps us to determine the predictive power and the nature of the numerous inter-relationships of the latent variables in the model and thereby evaluate the hypotheses provided in the theoretical framework. The evaluation of the structural model is undertaken in accordance with the method outlined by Hair et al. [96]. In the first step, an Algorithm PLS analysis is performed to assess the degree of collinearity between the predicted constructs, with the VIF value kept below three [101].

The path coefficients of the established associations are then calculated by executing the bootstrapping procedure in full mode with 5000 random subsamples and a 95% confidence interval. These coefficients, whose values range from 0 to 1, reflect the extent to which a change in the value of the exogenous variable affects the value of the endogenous variable. The R^2^ coefficients are then used to evaluate the predictive power of the model for each variable. According to Hair et al. [96], R^2^ values of 0.25, 0.50, and 0.75 are weak, moderate, and significant, respectively.

In the subsequent analysis, the omission distance D was determined by the constraint that the sample size cannot be divided by this number to yield an integer. Consequently, the D value selected was 8 (sample size = 196). According to Hair et al. [96], the significance and importance of the relationships, collinearity, and the value of the coefficients of determination (R^2^) must be evaluated. The direct and indirect effects of doing the bootstrapping technique in full mode with 5000 random subsamples are shown in Table 3 and Table 4, respectively.

**Figure 2:**
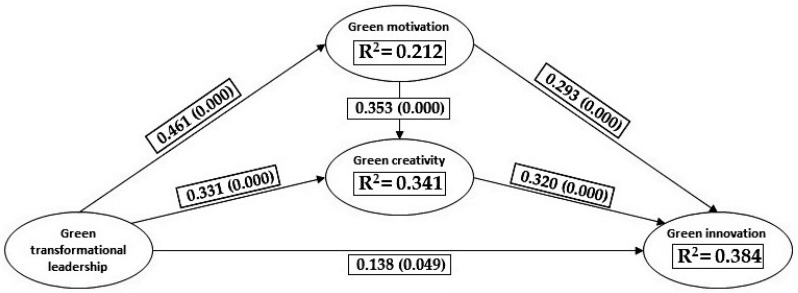
Nomogram of the model: path and determination coefficients.

The data analysis indicates that there is no collinearity, as all VIF values are less than three [101]. Green transformational leadership has a positive and statistically significant effect on firms’ green innovation (0.138, *p* = 0.049). However, most of the effect is produced indirectly through the mediating variables “Green motivation” (0.135, *p* = 0.000) and “Green creativity” (0.106, *p* = 0.000). In addition, they both exert a double mediation effect (0.052, *p* = 0.000), so the capacity of green transformational leaders to drive personnel motivation and develop their creativity skills is established as a key element for the development of green innovations. The proposed model explains 21.2%, 34.1%, and 38.4% of the variance of the “Green motivation”, “Green creativity”, and “Green innovation” components, respectively. Then, the four hypotheses proposed are accepted. To make easier the understanding of the results, the path and determination coefficients are exposed in the nomogram of the model displayed in Figure 2.

## 5. Discussion and Conclusions

Our research offers a fresh viewpoint on how green transformational leadership can influence the firms’ capacity to develop green innovations, both directly and indirectly. The results show a positive and significant effect of green transformational leadership on green innovation (Hypothesis 1). However, besides a direct relationship between these two variables, it has been evidenced that there is an indirect influence, which is materialized through the green motivation and green creativity variables (Hypotheses 2 and 3). Moreover, these two mediating variables are related, establishing a double mediation between the variable’s green transformational leadership and green innovation, so the motivation of employees in environmental matters has a positive and significant influence on their level of creativity in the development of solutions to reduce the environmental impact or even favor its regeneration (Hypothesis 4).

Regarding the managerial implications of the results, this present study reveals the practice of the Spanish wineries and offers new insights into favoring the development of adequate contexts within organizations which drive their environmental leadership, motivation, creativity, and the development of green innovations, through which reduce the negative impact of the firms’ activity on the environment, or even its regeneration. It is worth mentioning that green transformational leaders deviate from conventional thinking patterns to safeguard the environment, all the while fostering innovation, encouraging employees to work on environmental concerns and motivating them to evaluate, from a critical standpoint, their current working techniques. In addition, they assist to highlight an unorthodox perspective, that is, green transformational leadership creates a culture in which employees are inspired to develop an open mind and see beyond the established, enhancing the efficacy of any firm’s environmental management system. Then, instead of solely focusing on the outcome, that is, the development of green innovations, firms may be mindful of the significance of establishing an effective green transformational leadership over employees, through which control over the whole process is needed to develop green innovations. This may include modifying organizational processes in order to drive employees’ motivation and creativity in environmental issues. 

Companies in the wine industry can adopt green transformational leadership practices by promoting environmental sustainability as a core value and inspiring employees to become agents of change in promoting sustainable practices. Leaders can encourage employees to participate in green initiatives, such as waste reduction, energy conservation, and sustainable sourcing of raw materials. Green transformational leadership can also involve collaborating with stakeholders, including suppliers and customers, to promote environmentally sustainable practices throughout the supply chain. Regarding green motivation, firms are able to motivate employees to engage in green practices by offering incentives for environmentally sustainable behaviors, such as providing rewards, recognition, or career advancement opportunities for employees who demonstrate a commitment to environmental sustainability. Employee training and development programs can also be implemented to promote green skills and knowledge. In this vein, green creativity may be promoted by encouraging employees to think creatively and innovatively about environmental sustainability, as well as through the establishment of innovation teams, involving employees from different departments, to explore new ideas and technologies that promote sustainability. The use of brainstorming sessions and creativity tools, such as design thinking, can also stimulate green creativity. Firms can increase their green performance by developing innovative solutions, such as sustainable packaging materials (biodegradable bottles or recycled materials) to reduce waste, and renewable energy technologies, e.g., solar panels, to reduce carbon emissions. They may also employ digitalization and automation of its production processes or restructure its organizations, establishing new structures that favor cooperation and interrelation between the different people and departments that make up the company.

The efficacy of an environmental management system is defined by managers’ responses to environmental issues and their use of transformational leadership to encourage staff [102]. Furthermore, we found that transformational leaders may influence the involvement of employees with organizational activities, which in turn motivates them to work by fostering a trustworthy atmosphere, and so favorably benefits the organizational performance. Fundamentally, the theory of social identity states that people feel compelled to identify with the norm of the leaders when they last integrate environmentally valued characteristics and possessions. Then, employees contribute to promoting the efficient and effective functioning of the organization through positive behavior when managers within the company are respected and admired as transformational leaders [103]. Managers that stimulate staff yield a beneficial effect on the green motivation and creativity of the whole firm. Green transformational leaders help employees to rediscover and develop their curiosity and creativity to produce original, creative, and feasible solutions for environmental concerns and to address environmental problems. Social and environmental aspects are factors that influence employees’ actions [104]. A leader’s primary problem is optimizing the discovery, development, and exploitation of essential resources and competencies inside the organization [105]. Therefore, green transformational leaders should try to foster employee motivation and creativity with all the resources at their disposal, including tools such as group dynamics, training appropriate to the skills and specialization of employees, or coaching sessions that foster a sense of belonging to the organization and a desire to improve the business and the natural environment among employees. Specifically, by emphasizing the crucial functions of green innovation, we provide further insights on how businesses that want to stay competitive can create value through the improvement of their environmental performance.

To generate ecologically sustainable and creative ideas, managers must provide an atmosphere in which green creativity is developed, nurtured, and rewarded. In such a setting, new behaviors must be fostered, and workers must have the autonomy to make their own judgments. Therefore, it is essential that company managers with the necessary skills acquire the role of green transformational leaders in order to inspire new values, ambitions, and behaviors among employees linked to the environmental sustainability of business activity, with the aim of encouraging their green motivation and creativity, which, according to the results of this study, have a positive impact on the development of green innovations. Furthermore, this may increase the green knowledge base of the company, driving its capacity to absorb new knowledge to be applied to the development of new products and processes to reduce the environmental impact of business activities. Moreover, we suggest that firms should pay more attention to enhancing their employees’ motivation in environmental terms, as well as driving their creativity skills, which are key factors to improve firms’ green innovation performance.

In connection with the theoretical implications, our study adds to the body of knowledge in the following ways. Previous research on the effect of green transformational leadership on green innovation has mainly yielded positive results. In this regard, Singh et al. [24] found that green transformational leadership positively influenced the green innovation of manufacturing firms in the United Arab Emirates. Similarly, Begum et al. [106] reported that green transformational leadership was positively associated with green innovation in Chinese manufacturing firms. These studies suggest that green transformational leadership can have a positive effect on green innovation by promoting environmentally sustainable practices within organizations. However, according to Soewarno et al. [107], the relationship between green transformational leadership and green innovation has been scarcely studied. In fact, to the best of our knowledge, to date, there are no studies that investigate the function of green motivation and green creativity as mediating variables on green transformational leadership and green innovation relationships. Thus, this study makes unique additions to the literature.

Previous research provides evidence of how leaders can promote sustainable practices within the firm by communicating a clear vision for environmental responsibility [108,109]. The employees’ sense of environmental responsibility and personal values can be a strong driver of sustainable behavior and innovation [110]. Moreover, organizations can also foster motivation for sustainability by providing employees with opportunities to participate in green innovation and sustainability initiatives and recognizing and rewarding sustainable behavior [111,112]. Furthermore, some studies showed that fostering a culture of creativity and innovation can promote green innovation and sustainability [7,113].

This research picks up the baton of previous research on the different constructs analyzed, proposing a model based on their findings that attempts to reveal the way in which the variables analyzed are related. This brings to the literature an approach based on the resource-based view theory to grasp and clarify how green transformational leadership, motivation, and creativity foster the development of green innovations by firms, thus contributing as well to the leadership, organizational creativity, and environmental management literature. In this way, the presence of well-established leaders in the company who promote values and attitudes of environmental sustainability among employees allows the generation of a new culture and an ecosystem favorable to the exchange of valuable ideas and the development of environmentally sustainable innovations, in which motivation and creativity act as fuels in the generation of green innovations.

The environmental decisions and understanding of green initiatives made by green transformational leaders may impact the effectiveness of a company’s green practices, innovations, and performance. In addition, green motivation in this context refers to personnel who are strongly motivated by environmental concerns. For its part, green creativity refers to those that have a great talent for generating ideas for green solutions. As shown by the results, both are necessary for generating activities that protect the environment from the damages resulting from the production and disposal of traditional items. Thus, by analyzing their possible implications for green innovation, we deepen our knowledge of the determinants of green innovation. Therefore, their function in this area should be further investigated [24,111,114,115,116]. Then, we addressed how green transformational leadership promotes green innovation in the wine sector by employing green motivation and creativity. Furthermore, given that most wineries in Spain are classified as small- and medium-sized enterprises, they are operating in a lean and innovative environment that aids in compensating for their limited means. Then, encouraging green creativity amongst staff seems to be a natural fit that can increase their green innovation performance in an effective way. In this regard, our findings contribute to the discourse on green innovation and small and medium enterprises [117,118,119].

When environmental sustainability becomes a strategic priority for society as a whole, it is vital that firms obtain the support and dedication of their management and employees to develop green innovations that contribute to reaching this goal. We conclude that business sustainability and development can no longer be attained by mass manufacturing and steady financial standing. Going green is the proper and only option for corporations under the Kyoto treaty, the Paris settlement, and other international environmental agreements. Thus, green transformational leadership promotes firms’ green innovation both, directly and indirectly, via green motivation and green creativity. In this vein, firms, management, and leadership should advise and inspire their staff to enhance environmental performance via green innovations, minimize the environmental impact of business activity, or even allow for the regeneration of the natural context.

The transition of the firms in the wine industry towards new production models that are more efficient and environmentally friendly must be addressed with the support of their managers and employees. Then, it is necessary for firms to have leaders who are well-positioned and aware of the importance of developing new products, processes, and organizations to increase the environmental sustainability of business activities. In addition, green transformational leaders should promote the alignment of employees’ motivation with green innovation targets, as well as drive their creativity skills, since they are necessary to reach this aim in an efficient and effective way.

In relation to limitations, this study has focused solely on the analysis of companies belonging to the wine industry. In addition, some other variables that may be relevant in explaining the green innovative performance of companies have not been considered, such as green intellectual capital, the degree of cooperation with relevant stakeholders, or pressure from the institutional sphere or the company’s customers. Based on these limitations, we propose as possible future lines of research the inclusion of these variables in the analysis of the green innovative performance of companies in order to try finding out the main factors that drive its development. Additionally, it could be interesting to extend this analysis to other industries which present different key success factors.

## Figures and Tables

**Table 1:** Assessment of internal consistency and convergent validity.

Internal Consistency and Convergent Validity				
	C.A.	rho_A	C.R.	A.V.E.
G.C.	0.870	0.873	0.902	0.607
Green innovation	0.714	0.717	0.875	0.777
G.M.	0.886	0.888	0.921	0.745
G.T.L.	0.868	0.871	0.901	0.603
External Loads	G.C.	G.I.	G.M.	G.T.L.
G.I. process		0.872		
G.I. product		0.891		
G.C. 1	0.783			
G.C. 2	0.779			
G.C. 3	0.845			
G.C. 4	0.801			
G.C. 5	0.710			
G.C. 6	0.750			
G.M. 1			0.909	
G.M. 2			0.846	
G.M. 3			0.840	
G.M. 4			0.856	
G.T.L. 1				0.766
G.T.L. 2				0.813
G.T.L. 3				0.713
G.T.L. 4				0.824
G.T.L. 5				0.806
G.T.L. 6				0.731
Vif	G.C.	G.I.	G.M.	
G.C.		1.518		
G.I.				
G.M.	1.270	1.459		
G.T.L.	1.270	1.436	1.000	

Note: C.A.: Cronbach’s alpha; C.R.: Composite reliability; A.V.E.: Average variance extracted; G.T.L.: Green transformational leadership; G.I.: Green innovation; G.M.: Green motivation; and G.C.: Green creativity.

**Table 2:** Evaluation of discriminant validity.

Discriminant Validity				
Fornell–Larcker	G.C.	G.I.	G.M.	L.C.
G.C.	0.779			
G.I.	0.537	0.882		
G.M.	0.505	0.519	0.863	
G.T.L.	0.493	0.432	0.461	0.777
HTMT	G.C.	G.I.	G.M.	L.C.
G.C.				
G.I.	0.677			
G.M.	0.568	0.651		
G.T.L.	0.556	0.546	0.523	

Note: HTMT: Heterotrait–Monotrait; G.T.L.: Green transformational leadership; G.I.: Green innovation; G.M.: Green motivation; and G.C.: Green creativity.

**Table 3:** Summary of direct effects.

Structural Path	Coef (β)	S.D.	*p*-Values	C.I. 95%	Results
G.C. -> G.I.	0.320 **	0.074	0.000	[0.166–0.477] **	
G.M. -> G.C.	0.353 **	0.073	0.000	[0.359–0.626] **	
G.M. -> G.I.	0.293 **	0.077	0.000	[0.581–0.762] **	
G.T.L. -> G.C.	0.331 **	0.072	0.000	[0.017–0.287] *	
G.T.L. -> G.I.	0.138 *	0.070	0.049	[0.136–0.438] **	H1✓
G.T.L. -> G.M.	0.461 **	0.063	0.000	[0.142–0.461] **	

Note: Coef (β): Path coefficient; S.D.: Standard deviation; C.I.: Confidence interval; G.C.: Green creativity; G.I.: Green innovation; G.M.: Green motivation; G.T.L.: Green transformational leadership; ->: Direct effect; ✓: Supported; ** Statistically significant at 1%; * Statistically significant at 5%.

**Table 4:** Summary of indirect effects.

Total Effect of G.T.L. on G.I.		Direct Effect of G.T.L. on G.I.		Indirect Effect of G.T.L. on G.I.			Results
Coef. (β)	T value	Coef. (β)	T value	Point Estimated		C.I. 95%	
0.431 **	7.044	0.138 *	1.966	Total	0.293		
				H2 = a_1_ × b_1_	0.135 **	[0.062–0.219]	H2✓
				H3 = a_2_ × c_1_	0.106 **	[0.047–0.184]	H3✓
				H4 = a_1_ × b_2_ × c_1_	0.052 **	[0.023–0.091]	H4✓

Note: Coef (β): Path coefficient; C.I.: Confidence interval; G.T.L.: Green transformational leadership; G.I.: Green innovation; ✓: Supported; ** Statistically significant at 1%; * Statistically significant at 5%.

## Data Availability

Data available upon request to the authors.

## Appendix A

**Concept**	**Items**	**Definition**
Green transformational leadership	GTL1	Green product development project leaders inspire project members with environmental plans.
	GTL2	Green product development project leaders provide a clear environmental vision for project members to follow.
	GTL3	Green product development project leaders get project members to work together for the same environmental goals.
	GTL4	Leaders of green product development projects encourage project members to achieve environmental goals.
	GTL5	Leaders of green product development projects act with the environmental beliefs of project members in mind.
	GTL6	Green product development project leaders encourage project members to think green.
Green motivation	GM1	Our company sets green objectives, goals, and responsibilities for managers and employees.
	GM2	In our company, managers include targets linked to the achievement of green results in their appraisals.
	GM3	We offer green benefits (transportation/travel) instead of giving prepaid cards to buy green products.
	GM4	Our company offers rewards based on recognition of environmental management for staff (public recognition, awards, paid vacation, time off, and gift certificates).
Green creativity	GC1	Company members propose new green ideas to improve environmental performance.
	GC2	Company members propose new ways to achieve environmental objectives.
	GC3	Company members promote and defend new green ideas to others.
	GC4	Company members develop appropriate plans for the implementation of the new green ideas.
	GC5	Company members rethink new green ideas.
	GC6	Company members often find creative solutions to environmental problems.
Green innovation	Product	GIPR 1 The company chooses product materials that produce the least amount of pollution to carry out product development or design.
		GIPR 2 The company chooses product materials that consume the least energy and resources to carry out product development or design.
		GIPR 3 The company uses the least amount of materials to compose the product to carry out product development or design.
		GIPR 4 The company would deliberate with circumspection whether the product is easy to recycle, reuse, and decompose to carry out product development or design.
	Process	GIPC 1 The manufacturing process effectively reduces the emission of hazardous substances or wastes.
		GIPC 2 The company’s manufacturing process recycles waste and emissions that allow for treatment and reuse.
		GIPC 3 The manufacturing process reduces the consumption of water, electricity, coal, or oil.
		GIPC 4 The company’s manufacturing process reduces the use of raw materials.
Measurement	Likert scale (−3 = Strongly disagree; +3 = Strongly agree).	
